# Medical Opinion and Movement

**Published:** 1909-10-02

**Authors:** 


### Medical Opinion and Movement.

Dr. A. Purckhauer, of Breslau, has studied the effect of the specific treatment of syphilis upon the Wassermann reaction, and in the *Münchner Medizinische Wochenschrift* he gives the results of his observations on more than 800 cases. In these cases the "deflection of the complement" test, or Wassermann's reaction, had been instituted one or more times. From these tests he concludes that the proper treatment of the disease consists of intermittent but frequently repeated courses of treatment, whether by injection or inunction. The earlier and more energetic the treatment is pursued, the sooner does the reaction become negative. In late secondary or tertiary stages the reaction is much harder to influence by treatment than in the early stages. Leukoplakia, he states, is almost invariably of syphilitic origin, and is accompanied by a positive reaction. He is especially emphatic in regard to the necessity to continue the treatment during the secondary stage, whether there are any manifestations of the disease or not. All these injunctions are, of course, by no means novel, but it is satisfactory to find the orthodox tenets of treatment in such a disease confirmed and upheld by the latest scientific researches and tests.

---

Where should the incision be made for a retropharyngeal abscess? Such is the question raised by Dr. F. Rösling, of Tubingen, who strongly favours the external cervical incision instead of the more usual internal buccal. The objections raised against the latter are that it may cause a sudden outflow of pus into the respiratory passage, or at least there is the danger of a subsequent trickling of purulent matter causing an infective bronchopneumonia. Moreover, the evacuation may be insufficient, and a re-incision may be necessary. In cases of "cold abscess" of bony origin, or of an adenitis with suppurating and caseous glands, it is impossible to deal with the condition satisfactorily by the buccal route. This, however, is generally admitted, and in such cases a cervical incision would be chosen by the majority of surgeons. Dr. Rösling, however, advocates the cervical incision in all cases. The operation may be carried out in front of the stenomastoid, according to the method of Burckhardt, or the incision may be made posterior to this muscle, passing behind the vessels and nerves of the neck. This method was originally instituted by Chiene and is the one favoured by Dr. Rösling; it is simpler and there is less risk of injury to the vessels. In spite of all the arguments Dr. Rösling raises in support of this method of treatment, it must be recognised that it involves an operation of the "major" class in substitution for a much simpler and more rapid surgical intervention.

---

Reference was made some time back in these columns to the "recalcification" method of treatment for tuberculosis instituted by Dr. P. Ferrier, of Paris. The hypothesis upon which the treatment is based pictures tubercular disease as essentially a decalcifying process of the organism, and the chief weapon to combat the affection consists in the ability to limit and eventually destroy the foci of disease by a process of calcification. So convinced was Dr. Ferrier of the efficacy of this line of treatment that he has, with the help of charitable friends, instituted a gratuitous dispensary for the poor in Paris where tubercular patients can attend and carry out the treatment. One of the principles of the treatment is that the patients should be able to continue their employment and work according to their strength. The diet is directed with the view to prevent the introduction of acids into the system and as far as possible hinder their formation. All wines, beer, cyder, and liqueurs are prohibited. Fatty acids and butter must be as far as possible replaced by cream. Sauces, vinegar, oranges, old cheese are all interdicted. Not more than 200 to 300 grammes of bread and 300 to 400 grammes of lean meat are allowed per day. A carbonated calcic mineral water, such as Pougues-Saint-Léger, is directed to be taken early in the morning and half an hour before each meal, and three cachets a day containing 8 grains of each of the three following: Calcium carbonate, calcium phosphate, and calcined magnesia. An account is given of this dispensary and the treatment carried out there in *La Révue Médico-sociale*. The patients are described as being thin but vigorous and appearing to withstand well the ravages of the disease. The condition of the lungs is described as at first stationary and then improving little by little, with a steady progress towards sclerosis and emphysema. The experiment—for such it must be called at present—is highly interesting, and deserves careful watching by authorities in this country.

---

The use of leeches as a means of local depletion has to a large extent been supplanted by the more surgical and aseptic methods of scarification and cupping. These different methods are, however, by no means identical in their effects; leeches and cupping extract about the same amount of blood—*i.e.* 15 to 30 grammes—at the time of application, but in the case of the former the hæmorrhage continues often for some hours after their removal, amounting possibly to 100 to 200 grammes or more. They may, in fact, effect a general as well as a local depletion of the system. Haycraft has shown that the heads of leeches contain a substance, probably in the salivary glands, capable of preventing blood-coagulation in animals, and it is supposed that this substance injected into the wound inhibits coagulation and so favours continuation of the hæmorrhage. Dr. P. Emile Weil has studied the subject more recently, and he finds that the characters of the hæmorrhage after a leech-bite resemble in all respects those of a hæmorrhage of a hæmophilic. The blood comes from the wound drop by drop continuously without any tendency to coagulation. When a clot does form it is soft, non-adherent, does not retract, and the bleeding may continue under the clot. *In vitro* the blood shows markedly delayed coagulation, the duration varying from 40 minutes to even a day or more. The clots fail to retract and tend to dissolve again early. The addition of a couple of drops of blood serum (human or animal) accelerates coagulation and renders the blood normal in behaviour. According to Dr. Weil, this effect of the leech upon the blood is not entirely local. He finds in a number of cases examined that the coagulation time of the venous blood of the general circulation is prolonged to the extent of about five minutes, and that it possesses the other hæmophilic characteristics already mentioned in a less degree. Leech extract injected intravenously into rabbits produces all the pathological symptoms of a hæmophilic: hæmatomata, hæmarthrosis and severe hæmorrhages caused by the smallest injuries.

---

Hæmatemesis complicating the gastric crises of tabes is a somewhat rare occurrence, but a case which would seem undoubtedly to be of this nature has been reported recently by Kollarit, of Budapest. For the last few weeks of his life the patient was seized with repeated attacks of hæmatemesis, sufficiently severe in character to give rise to the suspicion of a gastric ulcer or of malignant disease. At the same time he passed blood in his stools. At the autopsy the characteristic lesions of tabes were discovered, but there were found no lesions of the gastric or the intestinal mucous membrane, nor could any other lesion be discovered capable of explaining the hæmatemesis. The case would therefore seem to have been one of parenchymatous gastric hæmorrhage accompanying a gastric crisis. A few cases of the kind have been described by Charcot, Vulpian, and Neumann, and Reichard has noted analogous cases occurring apart from tabes dorsalis. It follows, therefore, that a severe gastric hæmorrhage does not necessarily exclude the diagnosis of a gastric crisis.

---

Excellent results are reported by Drs. David and Iser in *Le Progrès Médical* from the local application of high-frequency currents in certain affections of the nose and pharynx. The local effect of these currents is threefold—analgesic, trophic, and bactericidal. The latter action may be produced by a coagulation of the protoplasm of the microbes, or by the production of ozone, or by the ultra-violet rays of the sparks. The conditions that have been treated by the authors in this way are atrophic rhinitis with ozæna, spasmodic rhinitis, and chronic dry pharyngitis. The current is applied to the affected mucous membrane by suitable electrodes. Applications were made three times a week at first, and then twice a week. The current was applied for five minutes, with very short sparks; but it was found better to use longer sparks, up to three or four centimetres, and diminish the time of application to only two or three minutes. The patients did not mind the treatment, and expressed themselves as feeling better and more energetic for work. Local reaction was shown by congestion of the mucous membrane, which, however, quickly disappeared. The authors have treated altogether seventeen patients, of whom twelve had atrophic rhinitis with ozæna, two spasmodic rhinitis, and three chronic pharyngitis. Of the twelve cases of ozæna, seven were completely cured, and the other five were markedly improved. In most of the cases the fœtor disappeared completely after the first applications. The dry crusts became moist and were discharged with removal of the electrode, but the treatment did not appear to have much effect upon the atrophy. Ten to fifteen applications were generally necessary to effect a cure. Both cases of spasmodic rhinitis and also the three cases of pharyngitis were completely cured after fifteen to twenty applications, although they had already exhausted all other forms of treatment, and were regarded as incurable. The authors seem justified therefore in their conclusion that the high-frequency currents offer a valuable means of treatment in the field of rhinological therapeutics.

---

A series of experiments with the new "S" bullet adopted by the German army has been carried out by Professor Fessler, of Munich, with a view to determining the nature of the injuries inflicted on the various body-tissues after being hit by such a projectile. From the report published in *Der Militaerarzt* it would appear that some 28,000 rounds of ammunition were expended, human cadavera and living horses being used as targets. The author finds that the flight of the projectile is much interfered with owing to the oscillation, and in about half the cases the bullet does not reach the quarry nose forwards, but sideways. Precision of fire and penetrative power are thus interfered with. In addition, terrible mutilation of the organic tissues occurs after a hit, so that healing is either impossible or much delayed. The author is therefore of opinion that great modifications must be introduced in order to render the bullet more accurate in its flight and less barbarous in its results.

---

Roubier, of Lyons, has recently published an interesting paper on the modifications which the blood undergoes in the various clinical forms of nephritis. Usually there is a diminution in the number of the red blood corpuscles, the anæmia varying in degree according to the variety of the nephritis, and also from time to time in the same case. This diminution in red cells is relative to the degree of œdema present, and varies according to the amount of dilution of the blood. At times no anæmia is present, and this is especially the case at the commencement of interstitial nephritis, when the number of red cells may even be increased. When anæmia is present there is usually a diminution in the hæmoglobin-content of the cells. Polymorphic red cells are only seen when the anæmia is intense. White cells are not increased in number when the nephritis is well compensated, but a slight leucocytosis may be present in cases where there is renal insufficiency or uræmia. It is the polynuclear leucocytes which are increased in this event. The author has noticed that the eosinophile cells tend to disappear from the circulation at the onset of uræmia. The coagulation period of the blood is usually normal, but in nephritis with hæmorrhage a slight delay occurs. In interstitial nephritis the colour of the blood serum is darker than usual. The serum may be opalescent or milky in some cases. The cryoscopic point is most commonly lowered. The amount of coagulable albumin in the serum is usually subnormal, and this is most marked when there is extensive œdema. In cardiac cases, however, this amount is frequently increased. The urea-content of the serum as a rule is raised in interstitial nephritis *pari passu* with the gravity of the case, but the chloride-content is normal. The hæmolytic power may be slightly raised, but in most cases is not altered. It will, of course, be understood that these various hæmatological characters vary somewhat according to the variety of the nephritis.

---

IN a recent number of the *Münchener Medizinische Wochenschrift*, Rabl publishes an account of four cases of strangulated hernia treated by subcutaneous injections of atropine. The cases were of varying degree of severity in persons differing widely in age, and in all cases the treatment was efficacious and its results prompt. The first case, that of a child, of three years, in which taxis had been tried without avail for the reduction of a strangulated inguinal hernia, proved amenable to an injection of three milligrams of atropine. An unpleasant sequence of this was the appearance of a violent delirium, with convulsions, dry skin, and tachycardia; but these symptoms were speedily overcome by a hot bath, followed by cold compresses to the head, and in two hours both the hernia and the delirium had disappeared. In the second case, that of a boy of twelve, in which taxis was impossible owing to pain, an injection of one milligram of atropine caused the reduction of the hernia in four hours. The third case was that of a man of 35, in which taxis had been tried without avail. An injection of six milligrams of atropine in the pubic region caused the hernia to disappear and relieved the pain; but the patient subsequently suffered from delirium and mydriasis. An old man of 81 with a strangulated scrotal hernia was the fourth case. An injection of three milligrams of atropine was given, but this augmented the pain, and a further injection of two milligrams of the same drug was administered five hours later. The hernia was subsequently reduced, but vesical paralysis and general debility followed, effects which might be fairly attributed to the drug, and the patient died a month later. The author claims to have never met with failure with this treatment. He recommends three milligrams of atropine as the minimum dose for an adult.

LOEPER and Gouraud have published in *La Presse Médicale* an article on the effects of a lime-free diet in cases of atheroma of the arteries. The authors maintain that, in addition to certain substances which are known experimentally to induce calcification of the arteries, such as oxalic acid, ergotin, tobacco, &c., some articles of food in general use which contain calcium are also capable of producing this condition. The arterial tissues have a special predilection for calcium, and animals which are fed on food containing an excess of lime-salts frequently suffer from calcareous degeneration of the arteries. The following are the foodstuffs which contain a large quantity of lime-salts: cow's milk, cheese, eggs, onions, beans, green cabbage, and strawberries. Lentils, haricot-beans, cauliflowers, and peas contain a moderate amount of lime-salts, whereas bread, meat, most fish, potatoes, asparagus, pears, and apples contain but little. Wine, beer, and perry are poor in lime-salts, whereas certain mineral waters contain much. In addition, as far as possible, to preventing the ingestion of lime, this substance should be eliminated by judicious use of drastic purgatives. Acids should not be given, for their administration helps to dissolve the lime fixed in bone and cartilage and to allow this to collect in the soft tissues, and consequently to favour the affection against which this treatment of Ferrier's was devised. The same disadvantages follow the use of iodides. From actual experiments on animals, the authors are led to believe that sodium bicarbonate is the best drug to use in the disease under discussion, as a notable diminution of the lime-salts in the cardio-vascular system has been found to follow upon its use.

---

AT the last meeting of the Italian Society of Medicine, Dr. Pagano, of Palermo, read a paper on the Mechanism of Functional Alterations in the Circulation in cases of Arterio-sclerosis. The author maintains that, in addition to functional disturbances, due to want of elasticity in the vessel-wall and diminution of its calibre, by which increased work is thrown upon the heart, there is a third and, in some respects, a more important factor at work. This factor is the result of the mechanism whereby reciprocal reflexes between the heart and vessels, and between the vessels themselves, are maintained. By this mechanism any disturbance occurring in the peripheral circulation is felt in the heart, as well as in other parts of the circulation. The knowledge that such a system of auto-regulation exists may, to some extent, necessitate the throwing aside of certain physiological and clinical theories of long-standing, in which the heart and vessels have usually been considered as independent parts of the circulatory system. Moreover, the reflexes extend not only to the different parts of the circulation, but to other parts of the organism, especially the respiratory apparatus. The result of these reflexes varies qualitatively and quantitatively, according to the part of the circulation affected. For example, excitation of the renal arteries produces a marked elevation of the blood-pressure, whereas excitation of the carotids reacts upon the heart, at times even stopping the beat. It is therefore easy to see that in all diseases which produce alterations in the walls of the heart and vessels, *e.g.* arterio-sclerosis, the auto-regulating mechanism undergoes functional disturbance which manifests itself in abnormal or exaggerated stimuli, starting from the arteries and showing their disturbing influences on the heart and other vessels. Such a mechanism may also be held to explain blood-pressure troubles in valvular disease and in toxic and infective conditions of the vessels, more especially if there be alterations in the arterial walls.

